# Proton and Carbon Ion Beam Spot Size Measurement Using 5 Different Detector Types

**DOI:** 10.1016/j.ijpt.2024.100638

**Published:** 2024-12-13

**Authors:** Matthias Witt, Uli Weber, Sebastian Adeberg, Kilian-Simon Baumann, Klemens Zink

**Affiliations:** 1 Institute of Medical Physics and Radiation Protection, University of Applied Sciences, Giessen, Germany; 2 Department of Radiotherapy and Radiation Oncology, Marburg University Hospital, Marburg, Germany; 3 Marburg Ion-Beam Therapy Center (MIT), Marburg, Germany; 4 Biophysics Division, GSI Helmholtzzentrum für Schwerionenforschung, Darmstadt, Germany; 5 LOEWE Research Cluster for Advanced Medical Physics in Imaging and Therapy, (ADMIT), TH Mittelhessen University of Applied Sciences, Giessen, Germany

**Keywords:** Spot size, Quality assurance, Pencil beam scanning, Detector comparison

## Abstract

**Purpose:**

The spot size of scanned particle beams is of crucial importance for the correct dose delivery and, therefore, plays a significant role in the quality assurance (QA) of pencil beam scanning ion beam therapy.

**Materials and Methods:**

This study compares 5 detector types—radiochromic film, ionization chamber (IC) array, flat panel detector, multiwire chamber, and IC—for measuring the spot size of proton and carbon ion beams.

**Results:**

Variations of up to 30% were found between detectors, underscoring the impact of detector choice on QA outcomes. The multiwire chamber consistently measured the smallest spot sizes, attributed to its intrinsic calculation model, while the IC array yielded larger spot sizes due to volume-averaging effects. These discrepancies highlight the necessity of selecting detectors based on QA needs, such as measurement speed, spatial resolution, and data acquisition methods. Digital detectors offer advantages over film-based ones by automating data processing, reducing manual errors, and providing immediate results.

**Conclusion:**

The study concludes that, although a single Gaussian fit is generally sufficient for QA, more sophisticated models might be beneficial for special applications. These findings aim to guide detector selection for ion beam facilities, enhancing QA procedures.

## Introduction

Pencil beam scanning (PBS) offers superior dose conformity to complex tumor geometries compared to traditional passive scattering techniques, making it the preferred method for proton and carbon ion therapy.^1^ Unlike passive scattering, in PBS, magnetically focused, narrow beams—typically millimeters to centimeters in size—are rastered over the target volume using scanning magnets.^2^ The spot size and shape are critical parameters that have a direct influence on dose distribution, treatment accuracy, and patient outcomes.^3^, ^4^, ^5^ These parameters are primarily determined by the optics of the beam transfer line, the extraction process, the scattering properties of the beam monitoring system (nozzle or snout), and the geometric distance from the beam exit to the reference point (eg, isocenter).

For low-energy beams and lighter ion types (eg, protons), the spot size and shape are predominantly influenced by multiple Coulomb scattering, resulting in Gaussian-like profiles, as described by Molière’s theory.^6^ In contrast, high-energy beams and heavier ions (eg, carbon ions) often deviate from this idealized distribution. These deviations arise from several factors, including nuclear fragmentation in heavier ions, nonlinear aberrations in magnetic fields used for focusing the beam, and chromatic effects caused by energy and momentum spread.^7^ The beam profile can further be altered when beam edges are truncated by beamline apertures such as septa or scrapers or by changes in the beamline optics.^8^ Due to the horizontal extraction process in synchrotron facilities, the beam shape of carbon ions is known to exhibit non-Gaussian beam shapes.^9^, ^10^, ^11^

Understanding and accurately characterizing these variations is critical for parameterizing input data into treatment planning systems (TPS)^12^ and establishing constancy measurements.^13^ Baseline parameter measurements during the TPS commissioning phase typically require high-resolution detectors and elaborate data analysis to establish the system's reference values.^14^, ^15^, ^16^, ^17^, ^18^ In contrast, quality assurance (QA) measurements prioritize efficiency, repeatability, and sensitivity to deviations, using detectors optimized for routine clinical workflows.^16^, ^19^, ^20^ The transition from commissioning detectors (eg, scintillation detectors or films as proposed in the AAPM TG-185 report on clinical commissioning of intensity-modulated proton therapy systems^17^) to routine QA detectors introduces challenges in ensuring data compatibility.

This study aims to address this gap by systematically comparing 5 detector types—radiochromic film, ionization chamber (IC) array, flat panel detector, multiwire chamber, and pinpoint IC. Each detector type offers distinct advantages and limitations, influenced by factors such as spatial resolution, measurement speed, and susceptibility to systematic errors like volume averaging. By examining variations in spot size measurements, we aim to determine which detectors offer accuracy, consistency, and suitability for QA in clinical settings. The results of this evaluation provide guidance on selecting detectors based on specific requirements, including measurement speed, spatial resolution, and data availability, to optimize QA practices and enhance treatment precision.

## Materials and methods

### Radiochromic film EBT3

**Figure 1:**
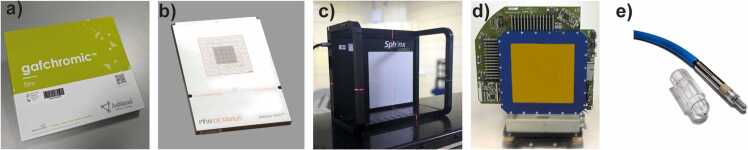
images of the 5 different detectors used in the study. From left to right (a) Gafchromic EBT3, (b) XDR1600, (c) Sphinx Compact, (d) ISO-MW, and (e) pinpoint chamber.

Self-developing radiochromic films have been an essential tool in radiotherapeutic QA for ion beam therapy since their introduction.^21^, ^22^, ^23^, ^24^, ^25^ Due to their high spatial resolution and dynamic dose range from 0.2 to 10 Gy, they can be used for dose verification and measurement of 2D dose distributions. In this study, the Gafchromic EBT3-film type (Ashland, USA) was used (Figure 1). The film consists of an active layer of 28 µm thickness sandwiched between two 125 µm polyester layers, resulting in a thickness of 278 µm and an equivalent density of 1.2 g/cm³.^26^ All films used were from the same batch (lot No. 10032202). Depending on ion sort, energy, and spot size combination, we applied a maximum dose of 2 to 2.5 Gy over a period of approximately 0.5 to 2.5 seconds for carbon ions and 2 to 6 seconds for protons. A detailed description of the calibration and scanning procedure is given in the Supplementary Material.

### Ion chamber array OCTAVIUS 1600XDR

The OCTAVIUS 1600XDR (PTW, Germany) is an IC array consisting of 1521 vented plane-parallel ICs, each measuring 2.5 mm × 2.5 mm × 2 mm. In the central area (7.5 × 7.5 cm²), the chambers are spaced 2.5 mm center-to-center and 5 mm in the outer area (15 × 15 cm²) (Figure 1). The effective point of measurement of the chambers is 6.9 mm below the surface.^27^ All measurements were performed in the central area with the highest spatial resolution. The interval time can be adjusted in 4 steps (100, 200, 400, and 800 ms), and when combined with the 2 measurement ranges (high and low), this allows for dose rates from 0.4 to 4000 Gy/min to be measured. All measurements were performed in the high measurement range with the fastest repetition rate of 100 ms. For each energy and spot size combination, we applied a total of 4 × 10^8^ carbon ions and 1 × 10^10^ protons over a period of 5 seconds at the central position. This resulted in a cumulated maximum dose at the spot center of approximately 5 to 20 Gy for carbon ions and 1 to 5 Gy for protons, depending on the primary energy and spot size.

For evaluation, the time-resolved data were processed using in-house software. The software integrates the individual 100 ms dose frames over the entire beam-on time (approximately 5 seconds). The line profiles along the central chamber axes (horizontal and vertical) were used to calculate the spot size, as described in the “Profile extraction and spot size determination” section.

### 2D flat panel Sphinx Compact

The Sphinx Compact (IBA, Germany) is a flat panel photodiode detector with a field size of 20 × 20cm² comprising 1024 × 1024 pixels with a pixel pitch of 0.2 mm (Figure 1). The detector is used for routine QA, including spot size measurements in other PBS (carbon and proton) facilities.^28^, ^29^ The effective point of measurement was estimated to be in 8.5 mm water equivalent depth.^29^ The detector offers 6 different gain values (0.25, 0.5, 1, 2, 4, and 8 pF). The gain corresponds to the sensitivity of the detector and, therefore, to the maximum applicable dose. In this study, a gain of 0.5 pF was chosen as a trade-off between measurement time, application time, and signal-to-noise ratio. Furthermore, this is the gain setting that is best specified and analyzed, as it is the gain setting used in the QA routine. Depending on the ion type and primary energy, a maximum dose of 0.6 Gy for carbon ions and 0.1 Gy for protons can be applied at this gain value. The dependence on the dose rate for carbons in the range of 1 × 10^6^ to 6.5 × 10^7^/s and protons in the range of 5 × 10^7^ to 2 × 10^9^/s was found to be below 1% (see Supplementary Material), which is consistent with the findings in.^29^

For each energy and spot size combination, a maximum dose of 0.3 to 0.5 Gy in approximately 0.5 seconds for carbon ions and a dose of ∼0.08 Gy in approximately 1 second for protons were irradiated.

The data provided by the software are the 2D-pixel data in DICOM (or.OPG) format. To ensure comparability, the DICOM images were analyzed according to the methodology used for the other detectors (see “Profile extraction and spot size determination” section), rather than using the software provided by the manufacturer.

### Isocentric multiwire chamber

The isocentric multiwire chamber (ISO-MW) (Siemens Healthineers, Germany, Figure 1) is based on the measuring principle of a multiwire proportional chamber but without gas amplification. To obtain the position information, the electrodes consist of equally spaced parallel wires. The anode plane is sandwiched between 2 orthogonal cathode planes and is arranged at an angle of 45° to allow position measurements on the x-plane and y-plane simultaneously. Each signal plane consists of 224 wires equally spaced every 1 mm. Each 2 neighboring wires are combined in 1 measuring channel, resulting in 112 measurement signals in x-direction and y-direction and an active area of 23 × 23 cm².^30^, ^31^, ^32^ A basic schematic of the internal components is given in the Supplementary Material. It is not possible to access the raw data of the ISO-MW. Instead, the manufacturer provides the already reconstructed position and spot size for each measurement cycle (∼100 µs). The high repetition rate is available because the ISO-MW is the same hardware as the position-sensitive monitors used in the beam application and monitoring system of the Siemens PT facility. These MWs feature a feedback loop to the scanner magnets for real-time position correction, which necessitates a high repetition rate.

The measurement data can be accessed in real-time via the TwinCAT system (Beckhoff Automation GmbH, Germany), or the data stored in the treatment records can be analyzed. The spot size determination algorithm is described in detail in the “Profile extraction and spot size determination” section. The beam plan and, therefore, the application time and dose were the same as for the XDR1600.

### Pinpoint chamber measurement

A set of 8 pinpoint chambers (PPCs) TM31015 (PTW, Germany) (Figure 1) was placed in a specially designed chamber holder, which was attached to a water phantom (MP3-P, PTW, Germany) used for patient-specific QA. The PPC is a small-sized IC used routinely for dose measurements. The chamber’s nominal sensitive volume is 0.03 cm³ with a radius of 1.45 mm. To facilitate measurements in air, the entrance window of the water tank was removed. The chambers are placed without any additional surrounding material, such as a built-up cap or support material. The mechanics of the water phantom were used for precise positioning and movement of the chamber holder across the beam. In that way, spot size measurements were performed for a few selected energy and focus combinations. The MP3-P axis allows movement in increments of 0.1 mm. For each energy and spot size combination, a maximum dose of 1.1 to 1.3 Gy in approximately 1 second for carbon ions and approximately 2 seconds for protons was irradiated. The irradiation was repeated multiple times for every position increment.

### Profile extraction and spot size determination

In this work, all spot sizes that are presented as the fullwidth at half maximum (FWHM) in millimeters are derived from a single Gaussian fit (Equation 1) and related to the Gaussian distributions σ by Equation 2. For the detectors, Sphinx Compact, Octavius XDR1600, and EBT3, 2 perpendicular line profiles (horizontal and vertical) through the center of the beam spot were extracted. For each line profile, a Gaussian distribution in the form of

(1) $$f\left(x\right)=a_{0}+A\;e^{-\frac{{\left(x-µ\right)}^{2}}{2\sigma^{2}}}$$

was fitted. With the parameters $a_{0}$ (constant offset), A (amplitude), µ (mean), and σ (standard deviation) to be optimized. The parameter $a_{0}$ is necessary to account for an offset in the data due to imperfect zero adjustment or background. The FWHM is then calculated by

(2) $$\mathrm{FWHM}=2\sqrt{2\ln2}∙\sigma$$

For the spot sizes measured with the PPC, the same approach was utilized with the exception that only horizontal profiles were measured.

Additionally, we also applied a double-Gaussian function to fit the measurement data:

(3) $$f\left(x\right)=A_{1}\;e^{-\frac{{\left(x-µ\right)}^{2}}{2\sigma_{1}^{2}}}+\;A_{2}\;e^{-\frac{{\left(x-µ\right)}^{2}}{2\sigma_{2}^{2}}}$$

The introduction of the second Gaussian, with the same center (µ) but with a different width (σ), is intended to address deviations from the zero-order term (single Gaussian) of the Molière function and account for the single-scattering tail^6^ and—in the case of carbon ions—the broader contribution from the fragments.

For the ISO-MW, the raw data are not accessible. Instead, the fully integrated software directly calculates and stores the spot size and spot position in treatment records. The calculation method of the FWHM is implemented according to Equation 4:

(4) $$\mathrm{FWH}M_{MW}=2\sqrt{2\ln2}∙s∙\sqrt{\frac{\sum_{i=x_{\mathrm{start}}}^{x_{\mathrm{end}}}\left(Q_{i}-C\right)∙\left(\left(i∙d\right)-{µ}_{\mathrm{Peak}}\right)²}{\sum_{i=x_{\mathrm{start}}}^{x_{\mathrm{end}}}\left(Q_{i}-C\right)}}$$

where s is an empirical scaling factor introduced by Siemens, d is the wire pitch (2 mm), $\left(Q_{i}-C\right)$ is the measured charge of channel i minus a constant Offset C, and ${µ}_{\mathrm{Peak}}$ is the position of the maximum calculated by

(5) $${µ}_{\mathrm{Peak}}=\;\frac{\sum_{i=1}^{n}\left(Q_{i}-C\right)∙i∙d}{\sum_{i=1}^{n}\left(Q_{i}-C\right)}$$

The constant offset is set to be 10% of the measurement signal. The empirical scaling factor s is, to our best knowledge, parameterized to the value 1.17.

### Beam delivery and data acquisition

All measurements were performed at the Marburg Ion Beam Therapy Center (MIT, Marburg, Germany).^10^, ^11^, ^33^ To cover a wide range of spot sizes, the combinations as presented in Table were chosen for this study. Five different predefined ion optics settings (labeled F1-F5) can be selected for the carbon ion spot size. The beam reference data in terms of spot size as a function of residual range as well as spot size as a function of distance to the nozzle are shown in the Supplementary Material. The different focus levels allow for an additional degree of freedom in the treatment planning process. Depending on spot size and spot distance, a steeper lateral fall-off or faster irradiation can be achieved.^16^, ^34^ The nominal spot sizes were set during the commissioning phase. All measurements were performed at the isocenter, which is located 1.4 m downstream of the beamline vacuum window. Therefore, the beam spot size for low carbon energies, as well as low-energy to medium-energy protons, is primarily influenced by multiple scattering, with beam optics and spot focusing playing a minor role. Consequently, the spot shapes for these ion types and energies are expected to follow Molière's theory of multiple scattering with a significant contribution in the single-scattering tail.^6^

**Table:** List of ion type, energy, and spot size combinations used in the study.

		Nominal spot size FWHM in air (mm)				
Ion type	Energy (MeV/u)	F1	F2	F3	F4	F5
Proton	48.08	32.5	-	-	-	-
Proton	80.69	19.6	-	-	-	-
Proton	132.65	12.3	-	-	-	-
Proton	157.58	10.7	-	-	-	-
Proton	221.07	8.1	-	-	-	-
Carbon	86.22	9.9	10.7	11.5	12.5	13.5
Carbon	149.96	6.5	7.6	8.7	10.0	11.3
Carbon	430.12	3.4	5.0	6.6	8.2	9.8

The listed spot sizes (F1-F5) are FWHM reference values in the isocenter used for commissioning of the accelerator and treatment planning.

**Abbreviation: FWHM, fullwidth at half maximum.**

For high-energy carbon ions and small focusing strengths (eg, large spot sizes), the beam shape is neither symmetric nor Gaussian shaped (Figure 5). This is caused by the extraction method and beam optics of the accelerator and is reflected at the isocenter because the multiple scattering is nearly negligible.

During the commissioning phase of the accelerator, a reference value for the spot size at the isocentre was determined for each ion type, energy, and focus level. This nominal value (Table) is used in the TPS for dose calculations. The reference value was initially measured using Kodak EDR2 film (which is not available anymore^35^) in the same setup as EBT3 films in this study. Since then, these values have been used as the reference value, and only relative deviations from these values are checked in QA.

All measurements presented were performed in 2 consecutive night shifts. The first measurement, as well as the concluding measurement of each shift, was performed with the Sphinx Compact as a constancy check. In addition, a second measurement session was performed with all detectors to verify the reproducibility of the results.

## Results

For all detector types except the ISO-MW, the presented profiles are displayed without any further postprocessing. The measurement results are shown in Figure 3 as the FWHM calculated by Equation 2 from the profiles by fitting a single Gaussian distribution (Equation 1).

As mentioned before, for the ISO-MW, the raw data are not provided, and therefore, a Gaussian fit to the beam profile cannot be performed. Instead, we use the provided $\mathrm{FWH}M_{\mathrm{MW}}$ (Equation 4) from the treatment records to calculate a Gaussian distribution with the same FWHM (Equation 2) and display this function.

**Figure 2:**
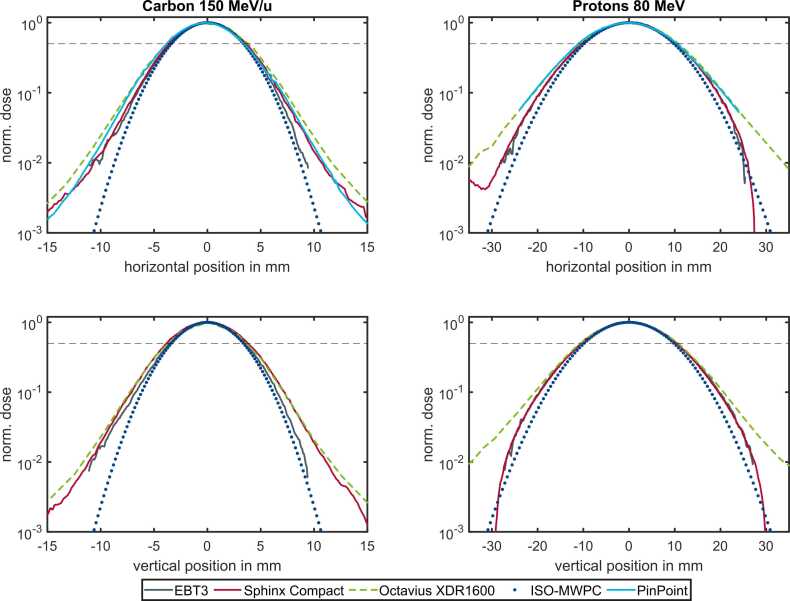
Normalized beam spot profiles measured with 5 different detectors. A 150 MeV/u carbon ion beam with a nominal spot size of 6.5 mm FWHM on the left side and an 80 MeV/u proton beam with a nominal spot size of 19.6 mm FWHM on the right side. The profiles are shown on a semilogarithmic scale. The horizontal direction is shown in the upper row, while the vertical direction is shown in the lower row. The different detectors are shown: EBT3 (solid gray), Sphinx Compact (dotted red), XDR1600 (green), ISO-MW (dark blue circles), and PPC (dashed-dotted light blue). The PPC measurement was only performed in the horizontal direction. The step size for PPC measurement was 0.4 and 0.5 mm for carbon and proton, respectively. The ISO-MW data are represented by a Gaussian curve, calculated via the measured $\mathrm{FWH}M_{\mathrm{MW}}$ (Equation 4). The horizontal dashed line indicates the 50% height of the profile corresponding to the FWHM for an ideal Gaussian. Abbreviations: FWHM, fullwidth at half maximum; ISO-MW, isocentric multi-wire chamber; and PPC, pinpoint chamber.

Figure 2 illustrates the beam spot profiles of 2 representative measured beams. The images in the left column display a carbon ion beam (150 MeV/u; 6.5 mm FWHM), while those in the right column show a proton beam (80 MeV/u; 19.6 mm FWHM). The spot size measured by the ISO-MW is the smallest across all profiles. As the ISO-MW profile is represented by an ideal Gaussian, it is evident that the profiles of the other detectors deviate from the ideal Gaussian shape, particularly at greater distances from the center. The profiles obtained with the EBT3 film are only shown for dose values above 0.01, as below these limits, the signal is indistinguishable from noise. For the proton beam there is a high level of agreement between the EBT3 and Sphinx Compact profiles over a range of up to 3 orders of magnitude. The differences between Sphinx Compact and XDR1600 are more pronounced for the carbon beam, especially at greater distances from the center. However, due to the single Gaussian fitting procedure, this difference is not reflected as clearly in the FWHM values. The horizontal profiles obtained with the PPC agree within 5% with EBT3, XDR1600, and Sphinx Compact. Furthermore, the non-Gaussian beam shape is also visible in those profiles. Both the contribution from large-angle scattered ions in the halo, as well as the slight asymmetry in the horizontal carbon ion profile, are reflected in all measured profiles.

**Figure 3:**
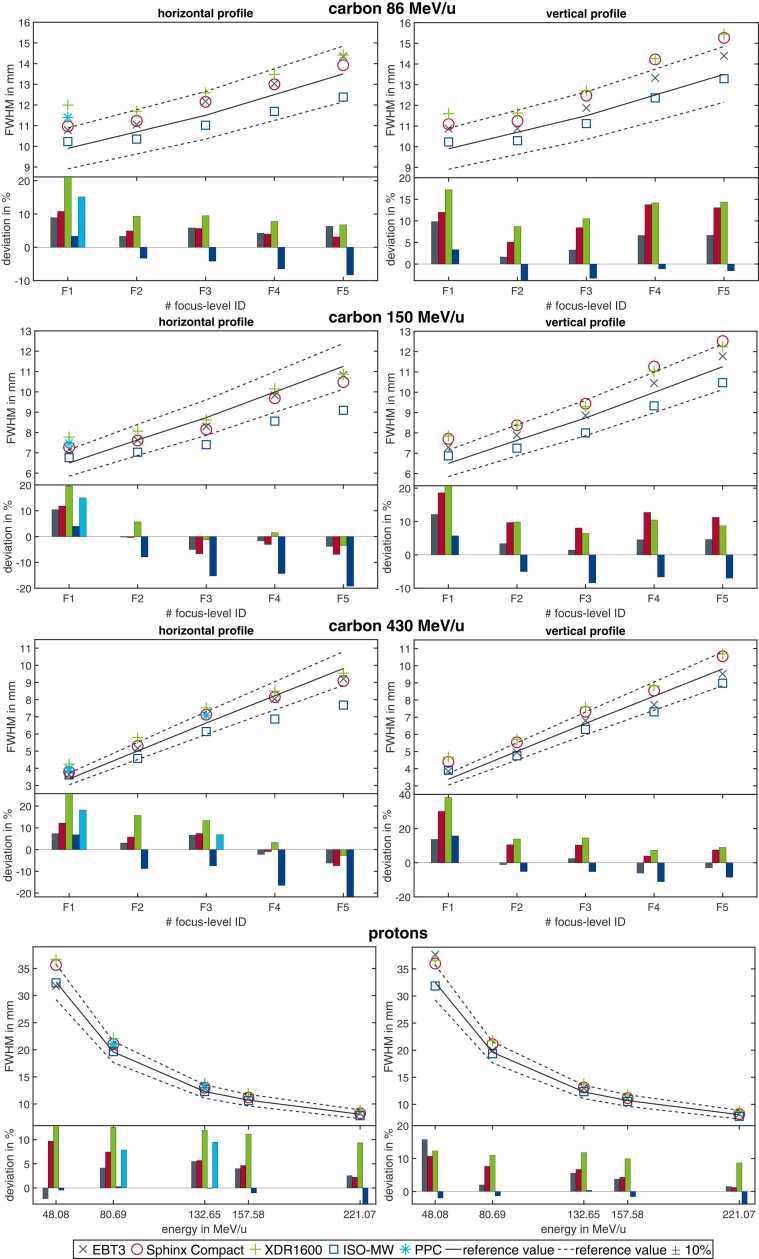
Measured FWHM values of the beam spot profiles in horizontal (left panel) and vertical (right panel) directions. For carbon ions (rows 1-3) and protons (last row). The reference values of the focus level IDs for each beam quality are given in Table. Each of the upper panels shows the measured FWHM (Equation 2) for the 5 different detector types: EBT3 (gray x), Sphinx Compact (red circle), XDR1600 (green plus), ISO-MW (dark blue square), and PPC (light blue star). The reference value used for the TPS base data calculations, with its tolerance level, is shown for visual guidance and is used to calculate a deviation for the bar plots in each corresponding lower panel. The colors of the bars correspond to the color of the marker for each detector. The PPC measurement was performed only in the horizontal direction and only for selected energy-focus combinations. Abbreviations: FWHM, fullwidth at half maximum; ISO-MW, isocentric multi-wire chamber; PPC, pinpoint chamber; and TPS, treatment planning system.

Figure 3 shows the measured spot sizes in the horizontal (left) and vertical directions (right) for all ion types, energy levels, and spot size combinations listed in Table. For visual guidance, the reference values with ±10% limits (QA tolerance) are displayed as solid and dashed lines, respectively. The ISO-MW recorded the smallest spot sizes across all profiles, with deviations of up to 20%. In contrast, the spot sizes measured with the XDR1600 were the largest for most profiles, with only 3 exceptions. The largest deviation, with a 38% larger spot size compared to the reference, was observed for the smallest applied focus. The spot sizes measured with the EBT3 film showed an agreement within 16% of the reference, while measurements from the Sphinx Compact showed deviations of up to 30%. In general, spot sizes measured with the EBT3, Sphinx Compact, and XDR1600 agreed within ±7% of each other, except for very small spot sizes. Furthermore, a better agreement between EBT3, XDR1600, and Sphinx Compact was observed in the horizontal direction than in the vertical direction.

**Figure 4:**
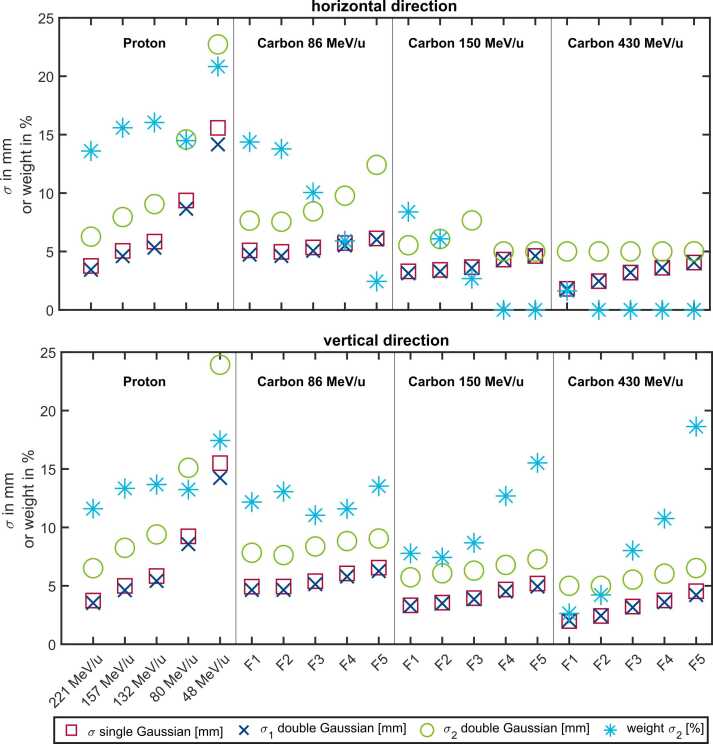
Results of the double-Gaussian (Equation 3) fit procedure of the profiles measured with the XDR1600. The left panel displays the results for the different proton energies in decreasing energy order. The carbon panels display the 5 different spot size reference values (F1-F5) for each energy (Table). The sigma as a result of the single Gaussian fit (Equation 2) is shown as squares. Sigma 1, sigma 2, and the weight of the second Gaussian (σ_1_, σ_2_, A2, Equation 3) are shown as crosses, circle, and asterisk, respectively.

In addition, a double-Gaussian fit (Equation 3) was performed on all profiles. The full table of results for every detector type (except ISO-MW) is given in the Supplementary Material. An excerpt of the double-Gaussian results for the XDR1600 is shown in Figure 4.

In the vertical direction (bottom panel of Figure 4), a double-Gaussian fit of the profiles improved the quality of the fit, as indicated by a lower root mean square error. However, for the horizontal direction, using the double-Gaussian fit did not enhance the fit quality for all combinations, in particular those with a sigma 2 weight of 0. For those specific energy, spot size, and ion combinations where the double-Gaussian fit did improve the fit quality, the contribution of the second Gaussian component was in the range of ∼10% to 20%.

## Discussion

The beam qualities of proton and carbon ion beams were measured using 5 distinct detector types. The spot size, as measured and fitted with a single Gaussian (Equation 1), showed significant variation depending on the detector used. The FWHM exhibited discrepancies of up to 38% compared to the reference value. A comparison among detectors showed that these differences ranged from −12% to a maximum of 31%. Even if the ISO-MW is excluded from the detector comparison, due to the different spot size determination algorithm and its included computational flaw (Equation 5), considerable discrepancies in the spot size from −6% to −30% remain. These deviations are much larger than the ±10% QA tolerances proposed by international protocols.^13^

### Non-Gaussian beam shape

Several factors contribute to the non-Gaussian beam shape observed. The first factor, previously mentioned, relates to deviations from the first-order term of Moliere theory due to large-angle scattered ions.^36^, ^37^, ^38^ This effect can be modeled by adding a second Gaussian term (Equation 3).

The application of a double-Gaussian fit yielded superior results for all proton beams. For carbon ions, however, this was not consistently the case across all beam qualities. Specifically, for higher primary beam energies and larger nominal spot sizes (ie, reduced focusing strength), the single Gaussian fit provided better results.

**Figure 5:**
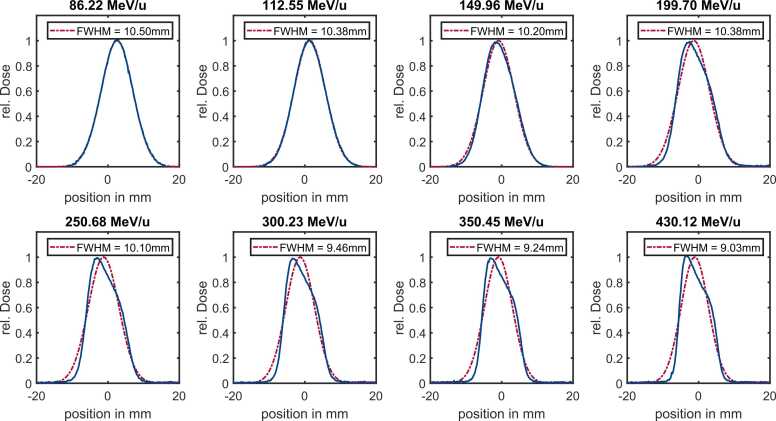
Film measurements of carbon ion spot sizes at the isocenter with a FWHM of approximately 10 mm and increasing energy. The blue line displays the measured horizontal profile, with the red dashed line fitted Gaussian distribution. The calculated FWHM value is displayed in the legend. As energy increases, the asymmetric beam profile becomes more pronounced. Abbreviation: FWHM, fullwidth at half maximum.

A second factor influencing non-Gaussian beam shapes is particularly evident in the measured profiles of higher-energy carbon ions (Figure 5). These profiles appear asymmetric and deviate from a Gaussian shape. This distortion is visible from beam exit to isocenter but is partially blurred by multiple scattering and due to the large distance from the nozzle. Consequently, the effect becomes more pronounced at higher energies, for heavier ions (such as carbon ions) and under weaker magnetic focusing (eg, larger spot sizes). Figure 5 displays carbon ion spot sizes of approximately 10 mm FWHM for different energies measured at the isocenter.

Using FWHM or σ as simplified measures of spot size constancy offers practical benefits. However, these non-Gaussian beam shapes cannot be fully captured by single or double-Gaussian models. For advanced applications such as TPS data acquisition or complex beam data descriptions, employing more sophisticated models like triple Gaussian or Gauss-Rutherford may be necessary.^12^, ^39^

### Spot size

The measured spot sizes showed large deviations. Some general observations can be drawn from the results.

The ISO-MW consistently measured the smallest values across all energy, spot size, and ion type combinations. This can partially be explained by the calculation model used. The empirical scaling factor, s, might be incorrectly parameterized (Equation 4) and the cutoff value, C, of 10% should not be subtracted. Using the cutoff value specifically removes contributions from large-angle scattering, leading to an underestimation of the spot size.

The XDR1600 showed the largest measured spot sizes for most of the profiles, with a clear tendency for small spot sizes to be broader. This can be partly attributed to a volume-averaging effect, given the comparable size of the ICs and the spot size.^40^, ^41^ A detailed description of the volume-averaging effect can be found in the Supplementary Material. Three of the 5 used detectors have a spatial resolution of 2 or 2.5 mm (XDR1600, ISO-MW, and PPC). For a nominal spot size of 3.4 mm, this leads to an overestimation of the spot size by up to 12.5%. It is important to be aware of this effect for detectors with a low spatial resolution.

The spot sizes measured with the Sphinx Compact showed good agreement with Pinpoint chamber TM31015 and the XDR1600, except for very small spot sizes. For the EBT3 film, the vertical direction consistently exhibited larger spot sizes than the horizontal direction, with no other systematic behavior, such as larger discrepancies for increasing or decreasing spot sizes or energy dependence, being observed. A directional dependence of the EBT3 film has already been reported by other groups.^42^, ^43^ However, as described in the Supplementary Material and according to established protocols,^44^ all films were handled to ensure uniform orientation during irradiation and scanning.

A directional dependence of the Sphinx Compact and XDR1600 was ruled out by performing successive measurements with detector rotation. A variation of <1% in spot size was observed when the detector was rotated along the beam axis.

The XDR1600, as well as the Sphinx Compact, have an effective point of measurement of 6.9 and 8.5 mm, respectively,^27^, ^29^ which is substantially deeper compared to the ISO-MW and EBT3 film. The additional scattering introduced by the extra built-up material is currently not considered.

### Repeatability and comparability of the data

Repeated measurements showed variations of <1% for each detector. The Sphinx Compact was used to verify spot size consistency at the start and end of each measurement shift, with all spot size measured within ±2% of the initial measurement. The 2% deviation includes changes from the beam application system as well as intrinsic detector errors. Measurements in immediate succession yielded deviations below 1%, for every single detector, while measurements 24 hours apart showed differences below ±4%. No systematic change was apparent when comparing these results. The Sphinx Compact and ISO-MW demonstrated the most stable detector responses with FWHM changes of ±1.5%, while the EBT3 film and XDR1600 exhibited deviations of ±3% and ±4%, respectively. This indicates that the much larger differences observed between detectors are systematic.

For long-term stability, we can primarily comment on the Sphinx Compact and ISO-MW, as these are the only detectors we use regularly. The XDR1600 was tested solely for QA purposes and is not available at MIT; hence, it is not used in clinical routine. Similarly, the EBT3 film, though recently tested for simultaneous spot-position and spot size measurement at ultra-high dose rates, is not regularly used. The ISO-MW has been used for spot size and spot position QA for over 10 years, and the Sphinx Compact has been part of the QA routine (2D homogeneity) since mid-2022. No radiation damage, aging effects, increased drift, or noise have been observed in either detector.

The high temporal resolution of the ISO-MW offers unique possibilities compared to other detectors. Since beam extraction in a synchrotron-driven machine is a dynamic process, the beam position and spot size of the beam can also show dynamic behavior over the extraction time. A mistuning of the machine may cause positional shifts or spot size variations throughout the extraction period. These potential errors could go undetected with static detectors or slower repetition rates.

In terms of the digital availability of the data, the EBT3 film has some major disadvantages compared to the other detectors. The EBT3 film requires complex protocols for scanning, handling, and calibration to ensure optimal results. Digital detectors streamline this process by automating data acquisition and processing, increasing accuracy, and reducing the potential for human error. Another challenge with EBT3 film is the mandatory postexposure waiting period, typically recommended to be at least 24 hours for accurate dose measurement,^23^, ^44^ whereas digital detectors provide immediate data availability, significantly speeding up the analysis process. However, with an adapted protocol (calibration and measurement films), a dose error of 1% could be achieved for films evaluated 30 minutes after irradiation.^45^

## Conclusion

The measured spot size is dependent on the detector used. Differences in measured spot size between the detectors exceed the ±10% QA tolerances recommended by international guidelines,^13^ highlighting that detector choice significantly influences measurement results and, consequently, the QA process. Typical differences in the order of 10% to 20% were measured; however, in extreme cases up to 30%. The spatial resolution of the detector should be selected to match the available spot size of the machine. Following the Nyquist theorem, errors should be reduced to an acceptable level. Employing a single Gaussian distribution for spot size QA as a measure of constancy appears sufficient, straightforward, and feasible for both carbon and proton beams. For specialized applications, employing more sophisticated models, for example, with double or triple Gaussian or additional exponential contributions, might further improve the agreement between fit and data.

## Declaration of Conflicts of Interest

The authors declare that they have no known competing financial interests or personal relationships that could have appeared to influence the work reported in this paper.

## Footnotes

Appendix A Supplementary material associated with this article can be found in the online version at doi:10.1016/j.ijpt.2024.100638.
