# Corrigendum to “Proton and Carbon Ion Beam Spot Size Measurement Using 5 Different Detector Types” [*Int J Part Ther.* 2025;15:100638]

**DOI:** 10.1016/j.ijpt.2025.100748

**Published:** 2025-04-23

**Authors:** Matthias Witt, Uli Weber, Sebastian Adeberg, Kilian-Simon Baumann, Klemens Zink

**Affiliations:** 1 Institute of Medical Physics and Radiation Protection, University of Applied Sciences, Giessen, Germany; 2 Department of Radiotherapy and Radiation Oncology, Marburg University Hospital, Marburg, Germany; 3 Marburg Ion-Beam Therapy Center (MIT), Marburg, Germany; 4 Biophysics Division, GSI Helmholtzzentrum für Schwerionenforschung, Darmstadt, Germany; 5 LOEWE Research Cluster for Advanced Medical Physics in Imaging and Therapy (ADMIT), TH Mittelhessen University of Applied Sciences, Giessen, Germany

The authors regret:

In the original version of the supplementary material, we identified two issues in Figure 4:

**Figure:**
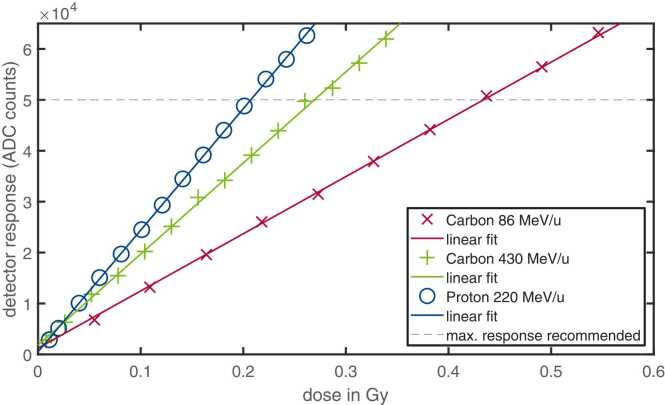

Incorrect y-axis label: The multiplier “×10⁴” on the y-axis label was inadvertently cropped in the published figure, leading to a misleading axis scale.

Incorrect slope for proton data (220 MeV): The slope of the curve for protons at 220 MeV was incorrect due to a missing scaling factor applied to the data. This has now been corrected by including the appropriate factor.

Improved readability: We have adjusted the marker size and line width in the figure to enhance visual clarity and readability.

File format change: The supplementary material is now provided as a PDF file instead of a DOCX file to ensure consistent formatting and appearance.

Both errors have been corrected in the updated figure. A marked-up version is provided alongside the corrected supplementary material for reference.

The authors would like to apologize for any inconvenience caused.

