# Supplementary material for: Corrigendum to “Proton and Carbon Ion Beam Spot Size Measurement Using 5 Different Detector Types” [Int J Part Ther. 2025;15:100638]
Source: Int J Part Ther. 2025 Apr 23;16:100748. doi: 10.1016/j.ijpt.2025.100748 (PMC12054106; doi:10.1016/j.ijpt.2025.100748)
Supplement: Supplementary file 1 — Supplementary material [file mmc1.pdf]

Supplementary Material

Calibration procedure EBT3

1. Dose-Calibration procedure

Since the dose response for the EBT3-films is not linear, a calibration procedure needs to be performed to convert the optical density (OD) to dose values. For film calibration homogeneous irradiation fields of 4x4 cm^2 with eight dose levels ranging from 0-5 Gy were used. The dose was checked with a calibrated ionization chamber (PTW TM31015), the homogeneity within the field was checked beforehand using the Sphinx-compact detector and was found to be within ±1.2%. Since the film has a slight dependence on the linear energy transfer (LET) [1], a wide range of LET values was used for the calibration procedure using protons and carbon ions at different energies (see Table 1 main document). For the calibration procedure, the films were irradiated at the isocenter with additional built-up material, resulting in a water equivalent depth of 7.5 mm. The additional material is required to match the setup used for dose measurements. However, for the spot-size measurements, the films were irradiated without the added build-up material.

2. Scanning procedure and calibration curve determination

All films were scanned using the Epson Expression 10000XL flatbed scanner. As recommended by the manufacturer, the films were scanned in transmission mode, at a color depth of 16 bit per channel and 300 dpi resolution. In this work, only the red channel was used for further analysis. Before the actual film scan, 50 scans were performed to account for temperature and heating effects. All films were scanned at least 72 hours after irradiation to minimize post-irradiation chemical processes [2]. Each film was placed at the center of the scanner using a positioning template and all films were scanned and irradiated in portrait orientation (as defined by [2]). A region of interest (ROI) at the center of the film with 50x50 pixels was analyzed. The optical density (OD) was calculated using:

$$OD = \log_{10}\frac{2^{16}}{P} \tag{1}$$

With P the mean pixel value within the ROI. The net optical density is then calculated by subtracting the OD of the unexposed (OD_{bg}) films:

$$netOD = OD - OD_{bg} \tag{2}$$

To fit the calibration curve the following formula was used [3]:

$$D(netOD) = a \cdot netOD + b \cdot netOD^n \tag{3}$$

with $D(netOD)$ being the dose at a given optical density $netOD$ for the selected scanner channel and a, b and n the parameters to be fitted. The obtained calibration curves are shown in Figure 1.

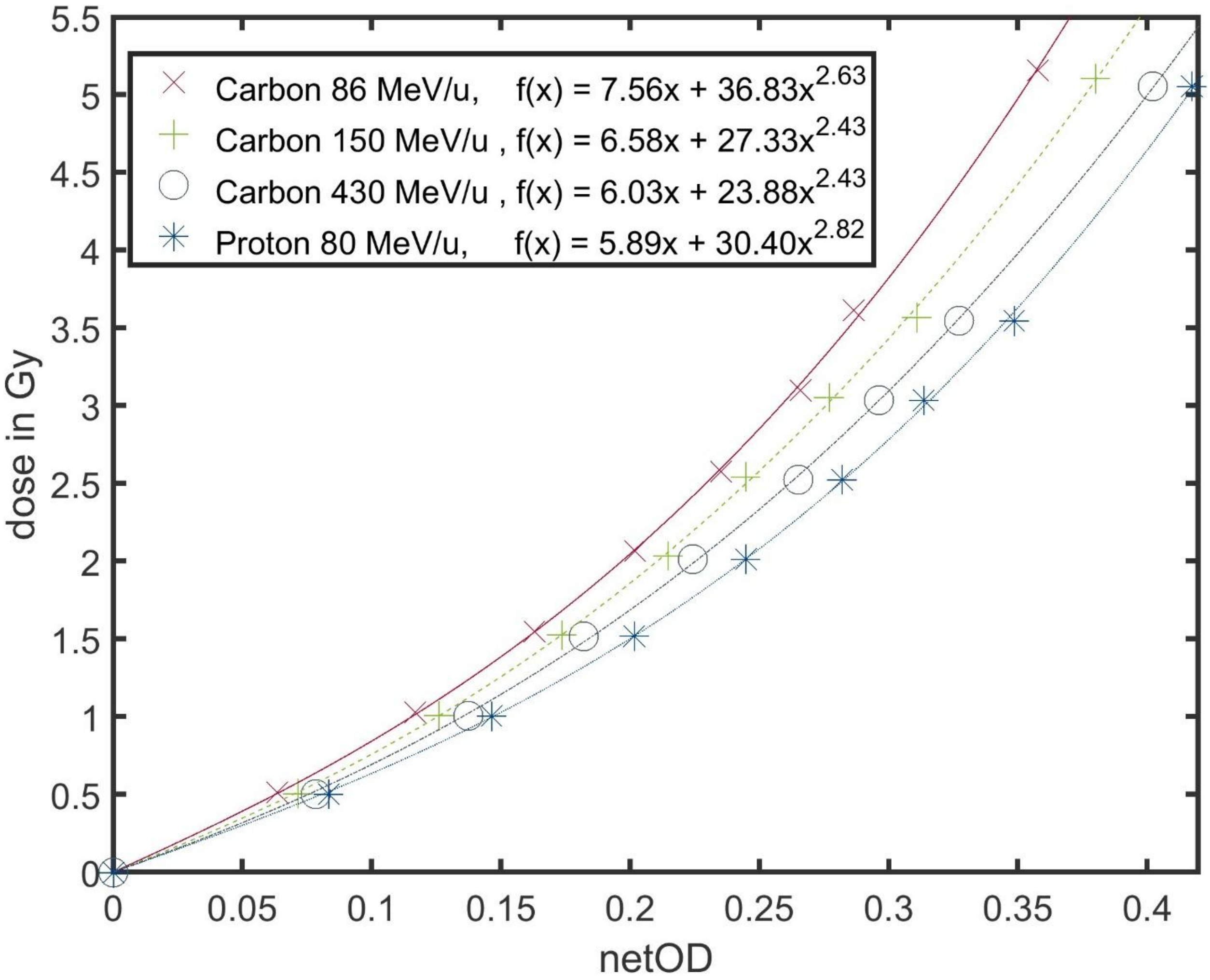

Figure 1: Calibration curves for the red channel of EBT3-film for four different ion type and energy combinations, resulting in different LET values at the measurement depth of 7.9 mm. With decreasing LET the steepness of the curve also decreases. The fitted calibration curve, along with its parameters a, b and n as defined in eq. (3) are provided in the legend.

The fitted calibration curve, along with its parameters a, b and n as defined in eq. (3) are provided in the legend. For all curves, the adjusted R^2 is higher than 0.9995. With increasing LET of the measured beam quality, a decrease of the steepness of the calibration curves is visible.

Schematic of multi-wire proportional chamber (MWPC)

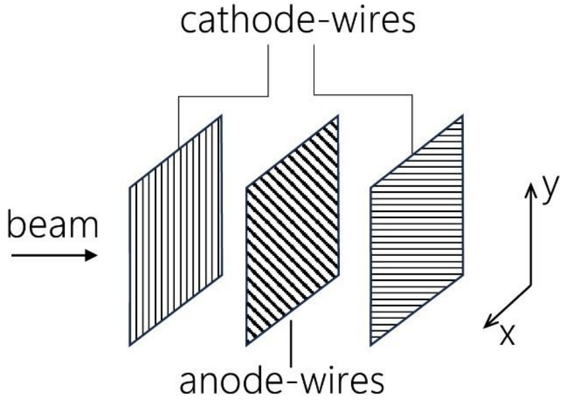

Figure 2: Schematic visualtisation of the anode and kathode wire orientation in the MWPC. The two kathode wire planes are rotated by 90° the anode wires are rotated by 45° to allow simultaneous measurement in both X- and Y-direction. Adapted from [4]

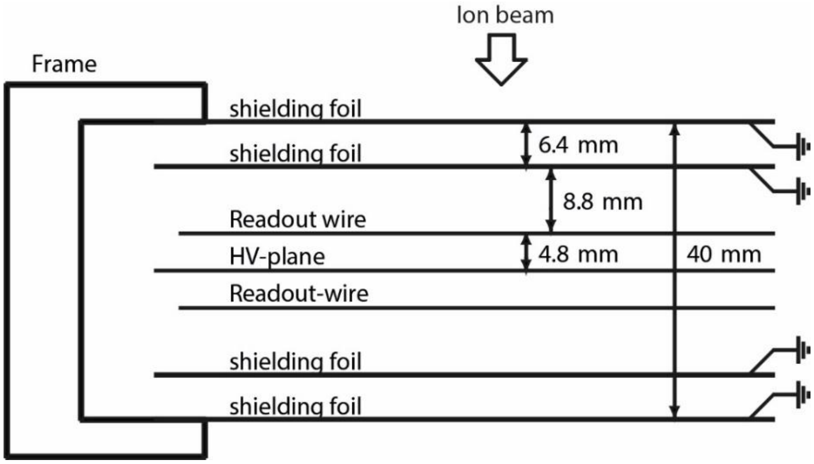

Figure 3: Schematic view of a MWPC taken from [5]

The outer boundaries of the MWPC are constructed from thin aluminum-coated Kapton foils. These 100 µm-thick foils are grounded and function as shielding against external electrical interference. Each readout plane consists of 224 parallel, gold-plated tungsten wires, spaced 1 mm apart, with a diameter of 20 µm[5]. The high-voltage (HV) plane is made of a metallized silk screen mesh. A detailed description of the functional principles can also be found in [6], [7].

If the ISO-MW is connected to the Siemens system, the measurement data is automatically included in the treatment records. The treatment records contain a horizontal and vertical position and spot size measurement for each raster-point. The given values are average values over the application time of each spot. For QA purposes, the application time of each spot can be manually adapted by changing the applied dose, applying dedicated dose rate or changing the measurement cycle time of the ISO-MW.

Sphinx Compact detector response functions

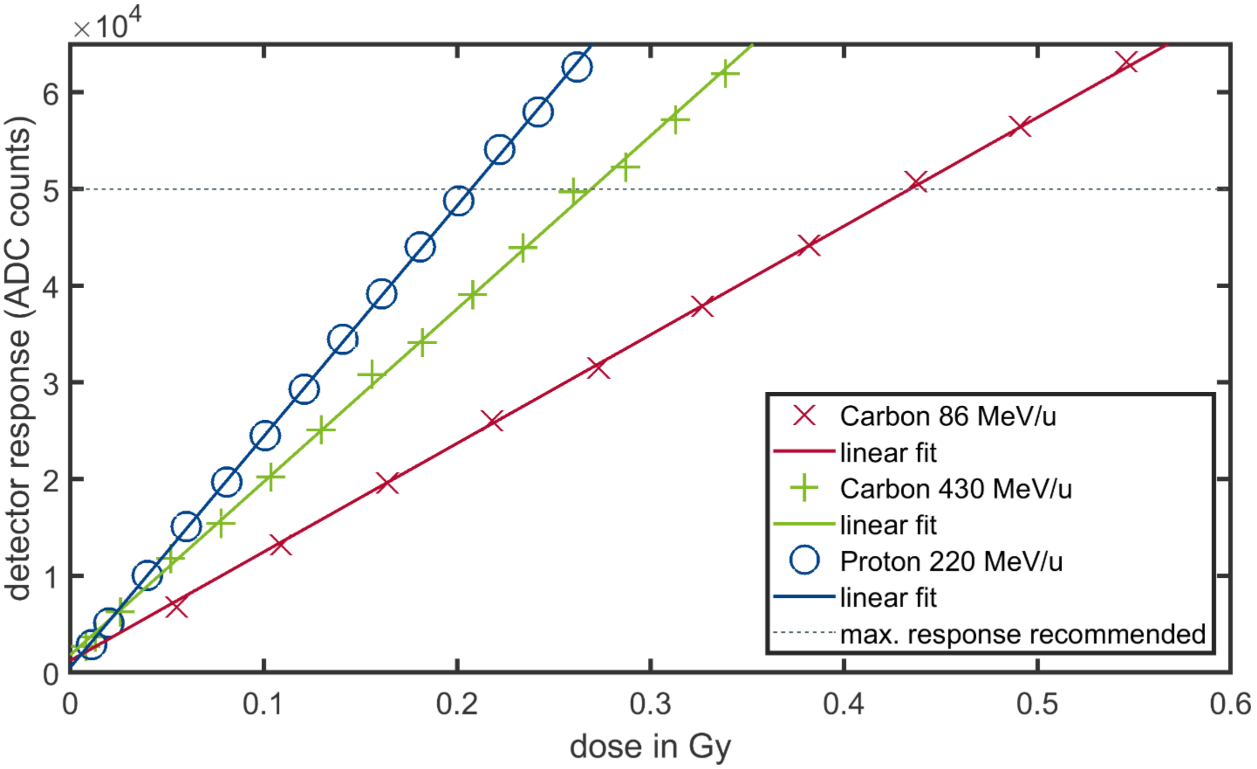

Figure 4: Detector response function of the Sphinx compact for the gain setting 0.5 pF.

Figure 4 shows the detector response function of the Sphinx compact for the gain value of 0.5 pF. For each of the three different energy and ion sort combinations, a 4 x 4 cm^2 field with 16 different dose levels was applied to the Sphinx compact. The detector response decreases with increasing LET. For all fitted curves the R^2 is above than 0.9991.

table 1: Dose rate dependency of the Sphinx Compact as percentage difference on the average signal in a homogeneous 4x4 cm^2 field obtained at different dose rates, relative to a reference dose rate (*), for a gain value of 0.5 pF.

Proton 221 MeV/u		Carbon 86 MeV/u	
Dose rate in ions / s	Difference from nominal dose rate*	Dose rate in ions / s	Difference from nominal dose rate*
5.00E+07	-0.35%	1.30E+06	-0.13%
6.90E+07	-0.26%	1.70E+06	-0.54%
9.70E+07	-0.56%	2.40E+06	-0.27%
1.30E+08	0.14%	3.40E+06	-0.46%
1.90E+08	-0.03%	4.70E+06	-0.18%
2.60E+08*	-	6.50E+06*	-
3.60E+08	0.18%	9.00E+06	-0.12%
5.00E+08	0.16%	1.30E+07	0.20%
6.90E+08	0.26%	1.70E+07	0.21%
9.70E+08	0.20%	2.40E+07	0.31%
1.30E+09	0.43%	3.40E+07	0.32%
1.90E+09	0.47%	4.70E+07	0.50%
2.19E+09	0.32%	6.50E+07	0.30%

In table 1 the percentage difference of the average signal in a homogeneous 4x4 cm^2 field relative to a value at a given reference dose rate (marked with *) is listed. The values were obtained with a gain setting of 0.5 pF. Within the 4x4 cm^2 field a ROI of 40x40 pixels (= 0.8x0.8 cm^2) was analyzed. The mean dose in the field was 0.4 Gy for carbon ions and 0.05 Gy for protons.

Beam reference data

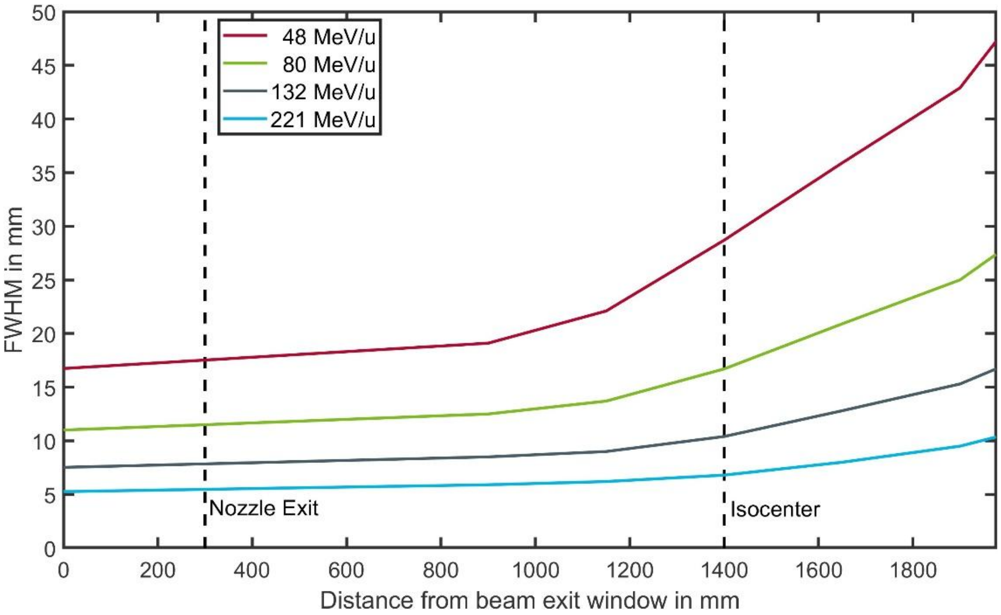

Figure 5: proton spot-size for four different primary energies as a function of distance from the beam exit window. The position of the isocenter as well as the last nozzle chamber are indicated by a vertical line. The values are taken from the beam reference data in the TPS and were originally measured with film.

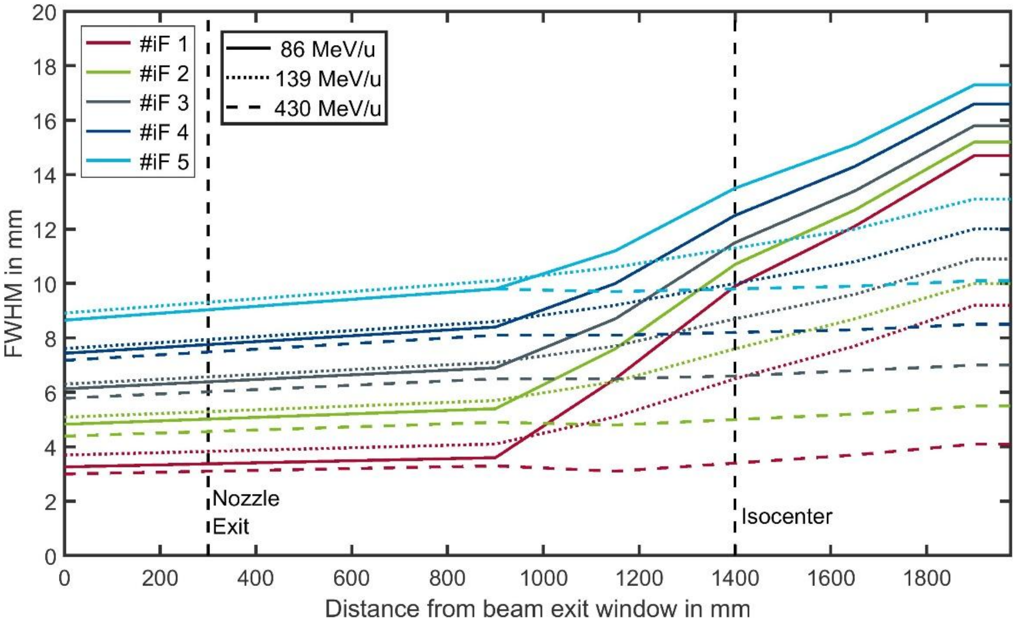

Figure 6: Carbon ion spot sizes for three different primary energies and five focus levels (#iF) are shown as a function from the distance from the beam exit window. The position of the isocenter as well as the last nozzle chamber are indicated by a vertical line. The values are taken from the beam reference data in the TPS and were originally measured using film.

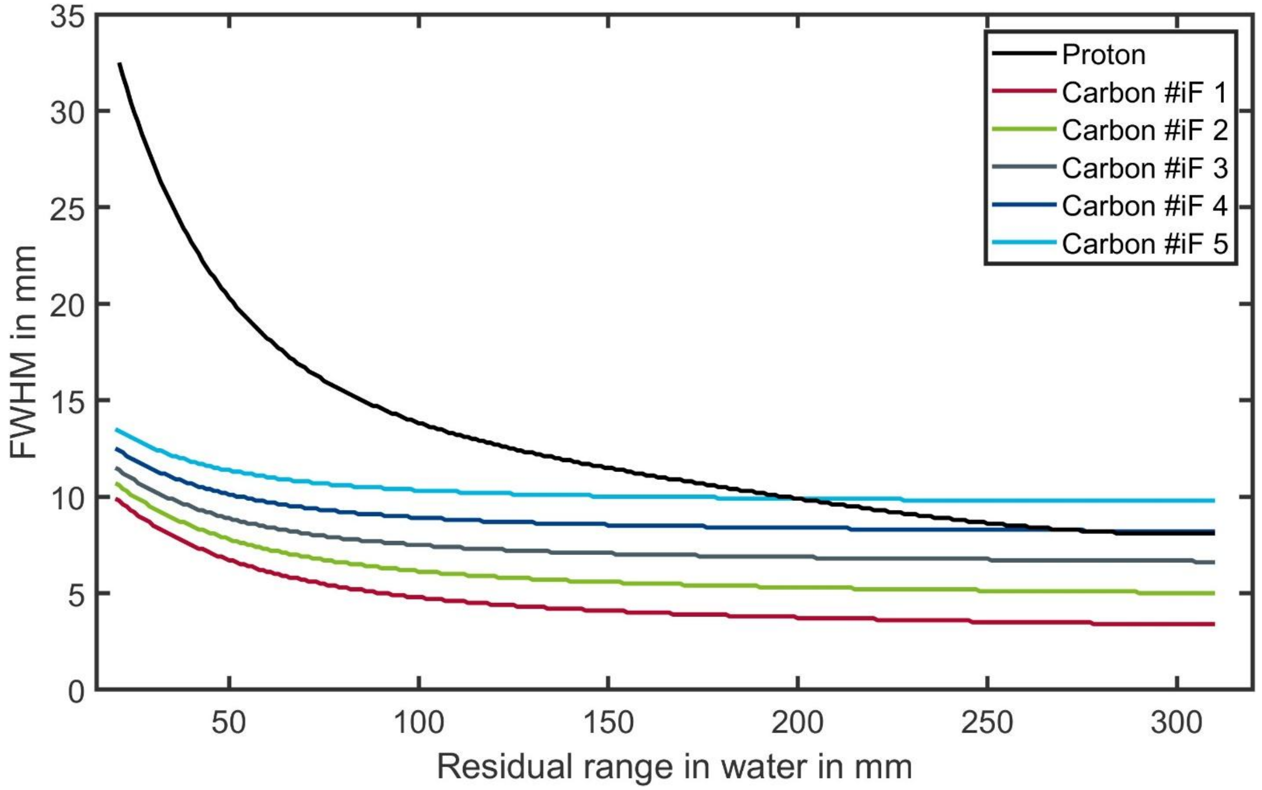

Figure 7: Reference values for spot sizes, for all energy and focus combinations of both carbon ions and protons, are presented as a function of the residual range in water. The values are taken from the beam reference data in the TPS.

table 2: results of double Gaussian fit procedure for EBT3 and Sphinx Compact. The weight of σ1 with its weight A1 and σ2 with its weight A2 for vertical and horizontal direction are given.

				EBT3								Sphinx Compact							
			reference value at isocenter	double Gaussian horizontal				double Gaussian vertical				double Gaussian horizontal				double Gaussian vertical			
ion type	Energy [MeV/u]	F#	σ [mm]	A1	A2	σ1	σ2	A1	A2	σ1	σ2	A1	A2	σ1	σ2	A1	A2	σ1	σ2
Proton	48.08	1	13.80	0.84	0.15	14.8	58.2	0.87	0.12	15.30	76.40	0.92	0.08	14.7	31.1	0.81	0.19	14.12	22.56
	80.69	1	8.32	0.86	0.12	8.8	51.3	0.86	0.13	8.66	38.32	0.93	0.06	8.7	21.9	0.86	0.14	8.42	14.51
	132.65	1	5.22	0.83	0.17	5.3	19.1	0.81	0.19	5.21	16.27	0.92	0.08	5.3	12.2	0.88	0.13	5.24	9.63
	157.58	1	4.54	0.83	0.16	4.5	16.8	0.84	0.15	4.51	17.86	0.91	0.09	4.5	9.3	0.90	0.11	4.47	8.59
	221.07	1	3.44	0.87	0.12	3.4	22.1	0.88	0.11	3.42	23.97	0.91	0.10	3.3	6.5	0.96	0.04	3.40	9.91
Carbon	86.22	1	4.20	0.84	0.14	4.4	20.8	0.85	0.14	4.50	22.92	0.91	0.09	4.5	8.4	0.89	0.12	4.46	7.60
	86.22	2	4.54	0.85	0.14	4.5	20.5	0.85	0.14	4.45	19.45	0.95	0.05	4.6	10.3	0.91	0.10	4.56	7.95
	86.22	3	4.88	0.85	0.15	5.0	23.9	0.84	0.15	4.91	24.55	0.97	0.03	5.1	15.3	0.91	0.10	5.09	8.51
	86.22	4	5.31	0.84	0.15	5.4	27.3	0.84	0.15	5.51	23.24	0.98	0.02	5.5	50.0	0.94	0.06	5.89	9.83
	86.22	5	5.73	0.87	0.12	6.1	50.0	0.83	0.16	5.89	19.09	0.99	0.01	5.9	49.8	0.84	0.17	6.18	8.70
	149.96	1	2.76	0.90	0.08	3.0	28.1	0.89	0.10	3.01	19.29	1.02	0.00	4.4	4.5	0.80	0.20	4.94	7.27
	149.96	2	3.24	0.91	0.08	3.2	32.1	0.89	0.10	3.25	18.73	1.01	0.00	4.1	4.1	0.80	0.20	4.45	6.40
	149.96	3	3.71	0.90	0.09	3.5	28.4	0.87	0.12	3.68	23.32	0.99	0.01	3.4	9.5	0.81	0.20	3.73	5.34
	149.96	4	4.24	0.93	0.08	4.2	50.0	0.86	0.13	4.29	19.09	0.96	0.04	3.1	6.2	0.88	0.12	3.37	5.16
	149.96	5	4.78	0.94	0.08	4.6	50.0	0.87	0.13	4.84	18.77	0.95	0.06	3.0	5.5	0.92	0.08	3.14	5.28
	430.12	1	1.43	0.96	0.06	1.5	50.0	0.92	0.07	1.63	50.00	1.04	0.00	1.6	39.6	0.97	0.04	1.82	3.39
	430.12	2	2.13	0.98	0.05	2.2	50.0	0.92	0.07	2.10	50.00	1.04	0.00	2.2	50.0	0.93	0.07	2.26	3.77
	430.12	3	2.82	0.99	0.05	3.0	50.0	0.92	0.07	2.88	50.00	1.04	0.00	3.0	3.1	0.80	0.20	2.84	4.35
	430.12	4	3.49	0.98	0.04	3.4	50.0	0.92	0.07	3.27	50.00	1.04	0.00	3.4	3.4	0.79	0.20	3.31	5.14
	430.12	5	4.17	0.99	0.04	3.9	50.0	0.91	0.08	4.03	50.00	1.04	0.00	3.8	1.0	0.79	0.20	4.10	6.36

table 3: results of double Gaussian fit procedure for XDR1600 and PPC measurement. The weight of σ1 with its weight A1 and σ2 with its weight A2 for vertical and horizontal direction are given.

			reference value at isocenter	XDR1600 double Gaussian horizontal				XDR1600 double Gaussian vertical				PPC double Gaussian horizontal			
ion type	Energy [MeV/u]	iF#	σ [mm]	A1	A2	σ1	σ2	A1	A2	σ1	σ2	A1	A2	σ1	σ2
Proton	48.08	1	13.80	0.80	0.21	14.2	22.7	0.83	0.17	14.24	23.92	-	-	-	-
	80.69	1	8.32	0.86	0.14	8.7	14.6	0.87	0.13	8.57	15.10	0.94	0.05	8.9	25.5
	132.65	1	5.22	0.84	0.16	5.3	9.1	0.87	0.14	5.42	9.40	0.73	0.26	5.2	7.8
	157.58	1	4.54	0.85	0.16	4.6	7.9	0.87	0.13	4.62	8.25	-	-	-	-
	221.07	1	3.44	0.86	0.14	3.4	6.3	0.89	0.12	3.53	6.51	-	-	-	-
Carbon	86.22	1	4.20	0.88	0.14	4.7	7.6	0.88	0.12	4.64	7.83	0.79	0.20	4.4	6.9
	86.22	2	4.54	0.86	0.14	4.6	7.5	0.87	0.13	4.65	7.63	-	-	-	-
	86.22	3	4.88	0.90	0.10	5.1	8.4	0.89	0.11	5.14	8.37	-	-	-	-
	86.22	4	5.31	0.94	0.06	5.5	9.8	0.89	0.12	5.79	8.83	-	-	-	-
	86.22	5	5.73	0.98	0.02	6.0	12.4	0.87	0.14	6.26	9.05	-	-	-	-
	149.96	1	2.76	0.92	0.08	3.1	5.5	0.92	0.08	3.27	5.73	0.85	0.15	2.9	5.0
	149.96	2	3.24	0.94	0.06	3.3	6.1	0.93	0.07	3.49	6.07	-	-	-	-
	149.96	3	3.71	0.98	0.03	3.6	7.7	0.92	0.09	3.85	6.30	-	-	-	-
	149.96	4	4.24	1.01	0.00	4.3	5.0	0.88	0.13	4.51	6.79	-	-	-	-
	149.96	5	4.78	1.02	0.00	4.6	5.0	0.85	0.16	4.94	7.27	-	-	-	-
	430.12	1	1.43	0.98	0.02	1.8	5.0	0.97	0.03	2.04	5.00	0.99	0.00	1.7	5.7
	430.12	2	2.13	1.03	0.00	2.5	5.0	0.96	0.04	2.44	5.00	-	-	-	-
	430.12	3	2.82	1.08	0.00	3.2	5.0	0.92	0.08	3.15	5.53	1.01	0.00	3.0	5.0
	430.12	4	3.49	1.06	0.00	3.6	5.0	0.89	0.11	3.59	6.04	-	-	-	-
	430.12	5	4.17	1.03	0.00	4.1	5.0	0.82	0.19	4.21	6.52	-	-	-	-

Volume Averaging effect on spot-size

The geometrical size of the chamber has an influence on the measured beam profile, especially for chambers with comparable size as the spot-size. This is caused by a volume averaging effect and can be mathematically described by a convolution:

$$P_m(x) = P_r(x) * K(x) \tag{4}$$

With $P_m(x)$ the measured profile, $P_r(x)$ the implicit real profile and $K(x)$ the detector response function [8]. In Figure 8 the effect of the volume averaging effect on the spot-size is displayed assuming a rectangular detector response function for a detector size of 2.5 mm.

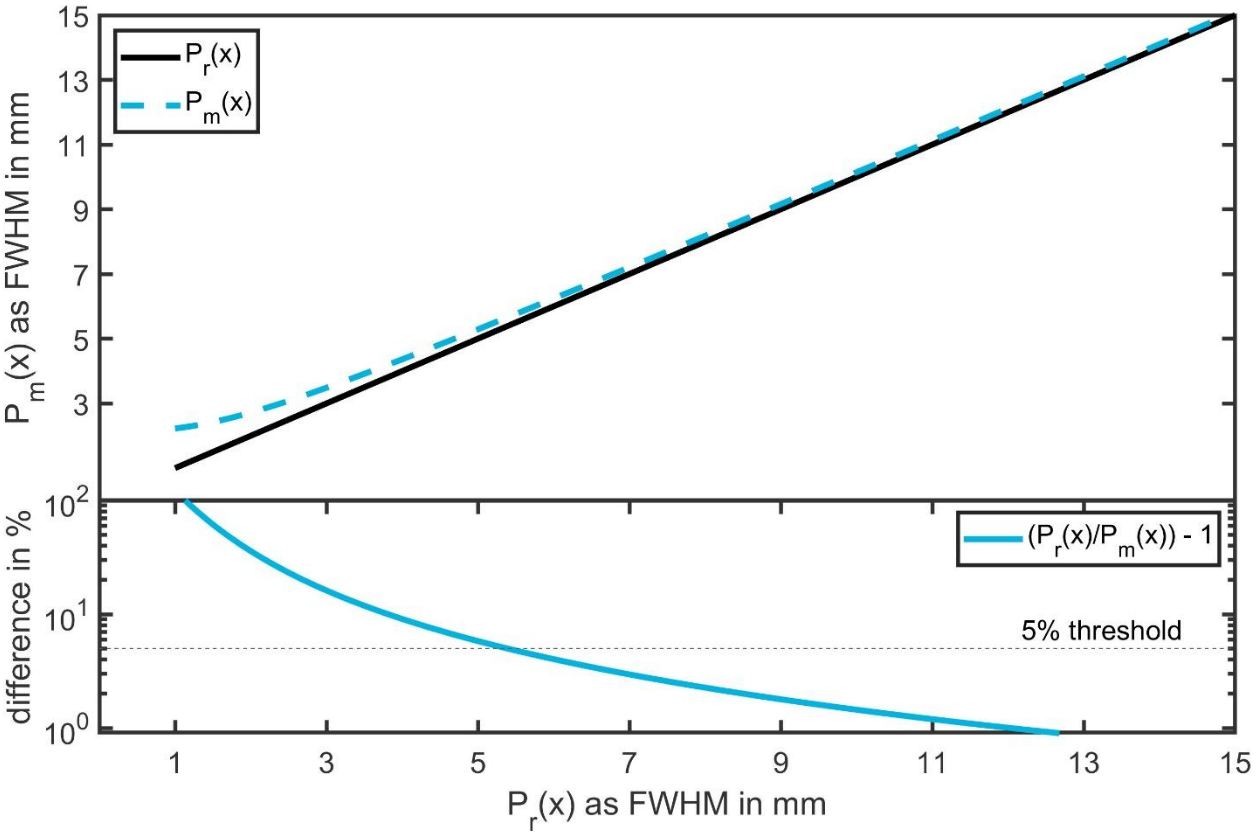

Figure 8: Impact of the detector size on the measured spot-size $P_m(x)$. In the upper panel the black line shows the implicit real profile ($P_r(x)$). The blue dashed line shows measured profile $P_m(x)$ by applying a rectangular detector response function $K(x)$. The volume averaging effect results in a larger measured spot-size, especially for spot-sizes with a comparable size to the detector. In the lower panel, the percentage difference is shown in a semi logarithmic scale. The dotted horizontal line indicates where the effect falls below 5%.
